# Durable engraftment of genetically modified FVIII‐secreting autologous bone marrow stromal cells in the intramedullary microenvironment

**DOI:** 10.1111/jcmm.13648

**Published:** 2018-04-23

**Authors:** Sze Sing Lee, Jaichandran Sivalingam, Ajit J. Nirmal, Wai Har Ng, Irene Kee, In Chin Song, Chin Yong Kiong, Kristoffer A. Gales, Frederic Chua, Edgar M. Pena, Bryan E. Ogden, Oi Lian Kon

**Affiliations:** ^1^ Laboratory of Applied Human Genetics National Cancer Centre Singapore Singapore; ^2^ SingHealth Experimental Medicine Centre Singapore Singapore; ^3^ Allpets & Aqualife Vets Pte. Ltd. Singapore Singapore; ^4^ Present address: Bioprocessing Technology Institute Singapore Singapore; ^5^ Present address: The Roslin Institute The University of Edinburgh Easter Bush UK

**Keywords:** autologous stem cell transplantation, bone marrow stromal cells, cell therapy, engraftment, factor VIII, gene targeting, haemophilia A, tissue microenvironment

## Abstract

Genetically modified FVIII‐expressing autologous bone marrow‐derived mesenchymal stromal cells (BMSCs) could cure haemophilia A. However, culture‐expanded BMSCs engraft poorly in extramedullary sites. Here, we compared the intramedullary cavity, skeletal muscle, subcutaneous tissue and systemic circulation as tissue microenvironments that could support durable engraftment of FVIII‐secreting BMSC in vivo. A zinc finger nuclease integrated human FVIII transgene into PPP1R12C (intron 1) of culture‐expanded primary canine BMSCs. FVIII‐secretory capacity of implanted BMSCs in each dog was expressed as an individualized therapy index (number of viable BMSCs implanted × FVIII activity secreted/million BMSCs/24 hours). Plasma samples before and after implantation were assayed for transgenic FVIII protein using an anti‐human FVIII antibody having negligible cross‐reactivity with canine FVIII. Plasma transgenic FVIII persisted for at least 48 weeks after implantation in the intramedullary cavity. Transgenic FVIII protein levels were low after intramuscular implantation and undetectable after both intravenous infusion and subcutaneous implantation. All plasma samples were negative for anti‐human FVIII antibodies. Plasma concentrations and durability of transgenic FVIII secretion showed no correlation with the therapy index. Thus, the implantation site microenvironment is crucial. The intramedullary microenvironment, but not extramedullary tissues, supported durable engraftment of genetically modified autologous FVIII‐secreting BMSCs.

## 1 INTRODUCTION

Haemophilia A treatment by protein factor replacement is invasive, expensive and only partially effective.^1^ Clinical outcomes of adeno‐associated viral (AAV)‐mediated gene therapy for haemophilia B and A are encouraging. However, there are unresolved challenges of AAV vector therapy. The most pressing are capsid‐specific T‐cell responses which reduce transgene expression and cause hepatotoxicity. Patients with pre‐existing antibodies may be unsuitable for AAV therapy, while treatment‐induced immune responses to AAV could preclude repeated treatment. Clinical scale vector production is costly and variable.

Genetically modifying autologous BMSCs for FVIII secretion for cell therapy could be a functional cure for haemophilia A. Bone marrow is an inexhaustible source of primary BMSCs which have a great capacity for ex vivo expansion. Naïve BMSCs physiologically secrete endogenous FVIII and can be genetically modified to express transgenic proteins durably in vitro and in vivo.^2^, ^3^, ^4^ A few studies have shown efficacy of naïve or genetically modified BMSCs implanted in haemophilic animals [reviewed in 5].

However, culture‐expanded BMSCs engraft poorly in vivo. BMSCs are intravenously infused in well over half of currently registered clinical trials. This is known to induce complement‐mediated BMSC destruction which may partly explain equivocal results from hundreds of BMSC clinical trials.^6^ The role of cell‐specific tissue niches has not been adequately investigated as a factor that determines survival and engraftment of implanted BMSC.^7^ We hypothesized that the microenvironment of the intramedullary bone marrow cavity would be more favourable than other tissue sites for FVIII‐secreting BMSC engraftment. Indeed, culture‐expanded human BMSCs engrafted and differentiated after intramedullary transplantation in NOD/SCID mice.^8^ Here we compare plasma FVIII levels after intramedullary, intramuscular, intravenous and subcutaneous implantation of autologous canine BMSCs modified to secrete FVIII by co‐transfection of AAVS1‐zinc finger nuclease (ZFN) and a donor human‐porcine FVIII transgene. Our data show highest and durable plasma levels of transgenic FVIII protein after intramedullary implantation, highlighting the importance of the implantation site microenvironment in cell engraftment.

## 2 MATERIALS AND METHODS

### 2.1 FVIII transgene integration in primary canine BMSC

Outbred adult male dogs were from the Agri‐Food & Veterinary Authority of Singapore. The study was approved by the Institutional Animal Care and Use Committee.

**Figure 1:**
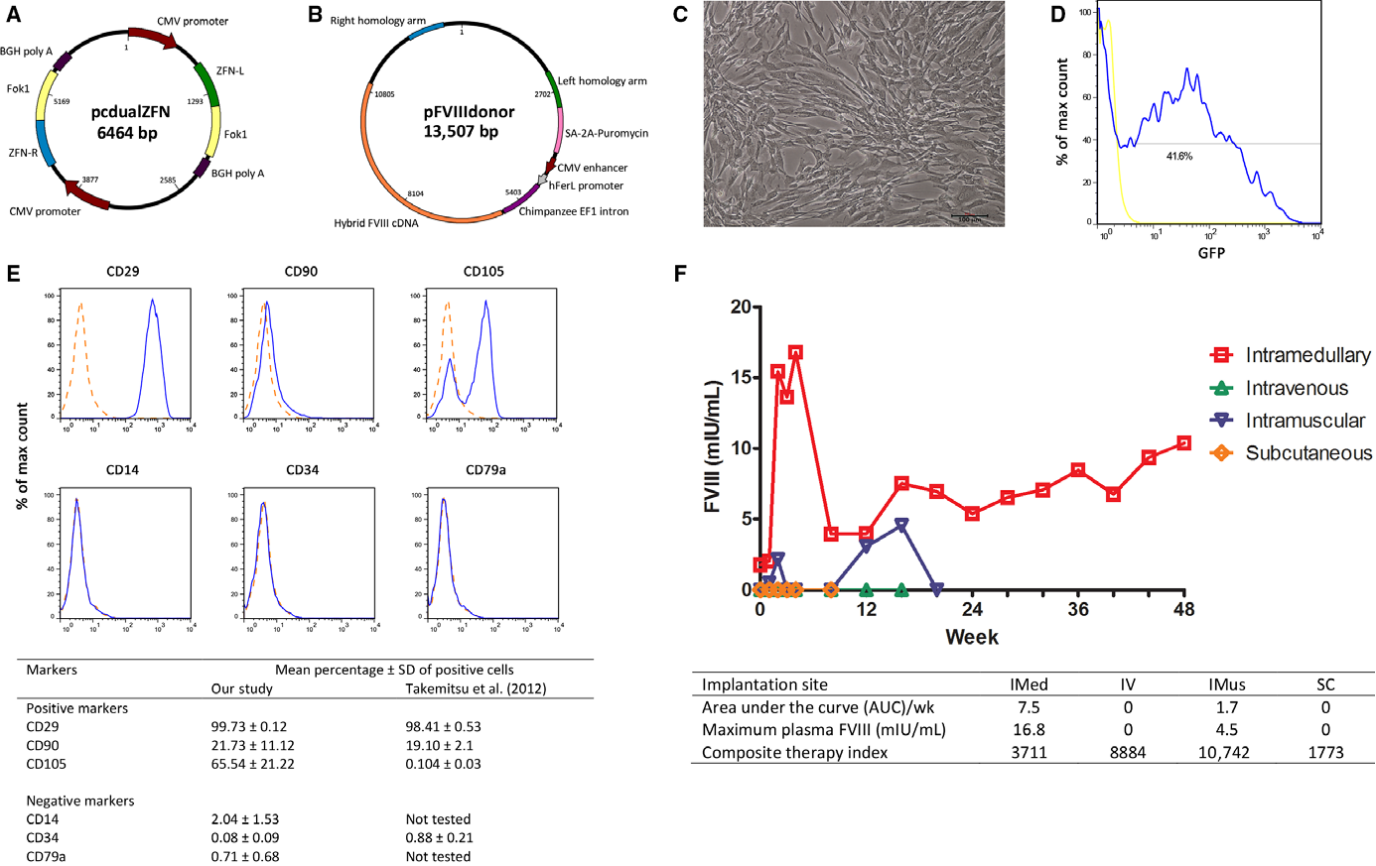
Maps of plasmids for ZFN‐mediated integration of donor FVIII transgene, immunophenotype of primary canine BMSC, and time course of plasma transgenic FVIII protein levels after autologous BMSC implantation in intramedullary and extramedullary sites. A, pcdualZFN expressed ZFN from dual expression cassettes of FokI endonuclease monomers and zinc finger peptides (ZFN‐L and ZFN‐R) targeted at canine PPP1R12C intron 1 (Sigma‐Aldrich, St. Louis, MO, USA). FokI monomers were modified for obligate heterodimerization and enhanced nuclease activity as previously described.^9^ B, pFVIIIdonor provided cDNA encoding a hybrid human‐porcine B domain‐truncated FVIII (5.1 kb) expressed from the human ferritin light chain promoter 10 flanked by 0.8 kb homology sequences at the ZFN target site. C, Primary canine BMSCs expanded ex vivo. Scale bar = 100 μm. D, Flow cytometry profile of GFP‐positive primary BMSCs of dog #5954 (intramedullary implantation) 24 h after electroporation with a GFP‐expressing plasmid. E, A representative profile of positive and negative markers of mesenchymal stromal cells present in early passage BMSCs in this study analysed by flow cytometry as previously described.^4^ Broken lines denote the corresponding isotype control. Bone marrow was obtained by aspiration through the trochanteric fossa of the femur. Summary of mesenchymal stromal cell markers of BMSCs of all dogs in our study compared with data of Takemitsu et al of primary bone marrow‐derived mesenchymal stem cells of 4 beagle dogs.^13^ The histogram subtraction method was used to calculate the mean percentage ± standard deviation of cells positive for each surface protein.^15^ All antibodies used for immunophenotyping were reactive against the cognate canine protein except anti‐CD105 antibody whose reactivity against canine CD105 was not specified by the manufacturer. F, Plasma levels of human FVIII protein after implantation of autologous ZFN‐treated BMSCs by the intramedullary method (IMed), intravenous infusion (IV), intramuscular (IMus) and Matrigel™‐encapsulated subcutaneous injection (SC). Summary of plasma human FVIII protein levels by implantation site and composite therapy index of each dog

All methods for primary BMSC culture, immunophenotyping and plasmid constructs for ZFN‐mediated integration of a FVIII transgene (Figure 1A‐C) were as previously described.^4^, ^9^, ^10^ ZFN‐modified autologous BMSCs were implanted in vivo without genetic selection after taking an aliquot for secreted FVIII activity assay. Parallel electroporation of a GFP plasmid was performed to determine the efficiency of gene transfer in each case (Figure 1D).

### 2.2 In vivo implantation

Autologous BMSC implantation was performed in sterile surgical conditions. We suspended cells in an equal volume of sterile phosphate‐buffered saline or Matrigel™ (final concentration 6 mg/mL; Corning Inc., USA). Intramedullary implantation was performed by injecting cells into the femoral medullary cavity. Intramuscular implantation was performed by injecting one‐fifth of the cell suspension into 5 different sites in the middle gluteal muscle. Matrigel™‐encapsulated cells were injected into fourteen subcutaneous sites (3 mL cell suspension/site) over the dorsal neck. Intravenous busulfan (6 mg/kg body weight; Otsuka Pharmaceutical Co., Japan) was administered 2 days before infusing cells into the cephalic vein.

### 2.3 Plasma FVIII and anti‐FVIII antibody assays

Citrated venous blood was drawn 3 weeks before cell implantation, immediately before implantation and thereafter at weekly intervals for 4 weeks, followed by monthly intervals. Plasma obtained by centrifugation (15 minutes at 1500 *g*; room temperature) was stored at −80°C until analysis.

Plasma samples were analysed for human FVIII protein using an antibody with negligible cross‐reactivity against canine FVIII (VisuLize™ FVIII antigen ELISA kit; Affinity Biologicals, Canada). All plasma samples were also tested for anti‐FVIII antibodies.^11^ FVIII activity in conditioned media of ZFN‐modified BMSCs was quantified as previously described.^10^

## 3 RESULTS AND DISCUSSION

Bone marrow‐derived mesenchymal stromal cells (BMSC) immunophenotypes in this study resembled that of human MSCs and previously described canine BMSCs 12, 13 (Figure 1E). Table 1 summarizes characteristics of in vitro BMSC expansion. The theoretical number of BMSCs expandable from all 10 mL of bone marrow aspirate from each dog was 1.5‐ to 20.2‐fold greater than the number of BMSCs used for FVIII transgene integration. These differences in canine BMSC yield and ex vivo expansion potential are similar to inter‐donor differences for human BMSC.

**Table 1:** BMSC growth characteristics and implantation of each dog, conditions of gene transfer by electroporation, gene transfer efficiency assayed by GFP plasmid expression and FVIII secretion in vitro

Dog ID	#5954	#5811	#6715	#5274
Implantation site	IMed	IV	IMus	SC
Bodyweight (kg)	25.5	20.2	18.0	23.5
Number of BMSCs grown from 10 mL marrow aspirate (P0 cells)	3.33 × 10^7^	7.48 × 10^7^	4.74 × 10^7^	15.6 × 10^7^
Cell expansion between passages (‐fold increase)	2.9‐4.1	2.8‐5.5	4.8‐10.2	2.5‐8.3
Number of BMSCs electroporated with pFVIIIdonor and pcdualZFN	2.3 × 10^8^	2.3 × 10^8^	2.2 × 10^8^	3.1 × 10^8^
Gene transfer efficiency^a^	41.6%	57.1%	84%	93.2%
Cell viability post‐electroporation	58.0%	80.5%	74.6%	41.3%
Number of viable BMSCs implanted	1.71 × 10^8^	1.34 × 10^8^	1.55 × 10^8^	0.57 × 10^8^
FVIII activity in conditioned media (mIU/10^6^ cells/24 h)	21.7 mIU	66.3 mIU	69.3 mIU	31.1 mIU
Composite therapy index^b^	3711	8884	10742	1773

BMSCs from fresh bone marrow were cultured in a 3:1 (v/v) mixture of low‐glucose Dulbecco's modified Eagle's medium (Invitrogen, Carlsbad, CA, USA) as previously described.^4^ BMSCs were expanded in culture for 3 weeks after bone marrow aspiration. The number of mononuclear cells in bone marrow aspirates was 3.54 × 10^8^ ± 1.21 × 10^8^ per mL (mean ± standard deviation; n = 4). The number of adherent cells which grew out initially after 9 days (P0) varied by 4.7‐fold among the dogs (3.33 × 10^7^ to 15.6 × 10^7^).

BMSC, bone marrow‐derived mesenchymal stromal cells; IMed, intramedullary; IV, intravenous; IMus, intramuscular; SC, subcutaneous with Matrigel™ cell encapsulation.

a Gene transfer efficiency measured by electroporating GFP (green fluorescent protein)‐expressing plasmid under identical conditions.

b Composite therapy index = FVIII activity in conditioned medium × number of viable BMSCs implanted.

FVIII activity was assayed in 24‐hour conditioned media of an aliquot of ZFN‐treated BMSCs taken on the day of implantation (Table 1). As the numbers of viable implanted BMSCs and secreted FVIII activities were variable among the dogs, a composite therapy index expressing the individualized “dose” of FVIII‐secreting cells implanted was calculated as the number of viable cells implanted × FVIII secretion in mIU/10^6^ cells/24 hours (Table 1).

Figure 1F shows the time course of plasma human FVIII protein levels in all dogs. Each served as its own control in calculating the difference between pre‐ and post‐implantation plasma human FVIII protein levels. Pre‐conditioning with busulfan to create a bone marrow niche was performed for intravenously infused BMSCs. Plasma human FVIII was undetectable at all time‐points after intravenous BMSC infusion and after subcutaneous implantation of Matrigel™‐encapsulated BMSCs. This was not because of an immune response against transgenic human FVIII as all plasma samples were negative for anti‐FVIII antibodies. In contrast, plasma human FVIII protein was detected after intramedullary and intramuscular implantation of autologous BMSCs. Expressing plasma human FVIII data as area under the curve (AUC) per week showed that intramedullary cell implantation (AUC/week = 7.5 mIU/mL) clearly achieved higher levels of plasma FVIII than intramuscular implantation (AUC/week = 1.7 mIU/mL). Using maximum plasma human FVIII levels as another indicator of efficacy, the peak plasma FVIII level of 16.8 mIU/mL after intramedullary implantation was also higher than the corresponding value after intramuscular implantation (4.5 mIU/mL). Although not as efficacious as intramedullary implantation, intramuscular implantation had a modest positive effect possibly because the muscle microenvironment provides some support for survival and engraftment of BMSCs which have a known potential for myoblast differentiation.

Prolonged secretion of transgenic FVIII after intramedullary implantation for at least 48 weeks with the bulk population of ZFN‐modified FVIII‐secreting BMSCs indicated that survival and engraftment of BMSCs had occurred in the perivascular niche whose relative hypoxia may favour cellular quiescence and stability. Like clinical cell therapy with hematopoietic stem cells, implanted ZFN‐treated BMSCs were not genetically pre‐selected for FVIII transgene integration.^14^ Nonetheless, successful BMSC engraftment in the intramedullary microenvironment enabled FVIII secretion from the unselected bulk population.

Our data showed no relationship between the composite therapy index and post‐implantation plasma transgenic FVIII levels. The composite therapy index for intramuscular implantation (10742) and intravenous infusion (8884) which showed modest or undetectable plasma human FVIII, respectively, were more than twice as high as the composite therapy index for intramedullary implantation (3684) which resulted in plasma samples with consistently highest plasma human FVIII levels for at least 48 weeks. Intravenously infused BMSCs were completely ineffective despite a high composite therapy index, consistent with rapid destruction by complement activation.^5^

Autologous cell therapy could be advantageous over factor replacement which is costly and requires frequent venous access. Cryopreserved BMSCs remain functional and can be used for repeat treatments. However, several improvements are essential for genetically modified autologous BMSC therapy to be clinically feasible. Techniques for transfecting primary BMSCs with non‐viral plasmid vectors which induce less cell death than electroporation are needed. More efficient transgene integration methods which do not greatly reduce cell viability would be preferable to ZFN. Whether the CRISPR/Cas9 technology could overcome these limitations is worth investigating. Despite these limitations, evidence of durable FVIII secretion after intramedullary implantation of autologous BMSCs in this study should encourage further attempts in this direction. Our data show the importance of the intramedullary microenvironment for engraftment of FVIII‐secreting BMSCs and advocate continuing efforts to develop genetically modified autologous BMSCs for haemophilia A treatment.

## CONFLICT OF INTEREST

The authors confirm that there are no conflicts of interest.

## AUTHOR CONTRIBUTIONS

Sze Sing Lee performed experiments, data management and manuscript writing. Jaichandran Sivalingam, Ajit J. Nirmal performed experiments; reviewed manuscript. Wai Har Ng performed experiments. Irene Kee, In Chin Song, Chin Yong Kiong, Kristoffer A. Gales performed veterinary management and procedures. Frederic Chua performed veterinary consultations. Edgar M. Pena performed veterinary oversight and assessment. Bryan E. Ogden performed veterinary procedures and oversight. Oi Lian Kon performed conception and design, data analysis, manuscript writing.
